# Whole genome analysis of echinocandin non-susceptible *Candida Glabrata* clinical isolates: a multi-center study in China

**DOI:** 10.1186/s12866-023-03105-3

**Published:** 2023-11-16

**Authors:** Yi Li, Xin Hou, Ruoyu Li, Kang Liao, Ling Ma, Xiaoming Wang, Ping Ji, Haishen Kong, Yun Xia, Hui Ding, Wei Kang, Ge Zhang, Jin Li, Meng Xiao, Yingxing Li, Yingchun Xu

**Affiliations:** 1 Department of Laboratory Medicine, State Key Laboratory of Complex Severe and Rare Diseases, Peking Union Medical College Hospital, Chinese Academy of Medical Sciences and Peking Union Medical College, Beijing, China; 2 Beijing Key Laboratory for Mechanisms Research and Precision Diagnosis of Invasive Fungal Diseases, Beijing, China; 3 Graduate School, Peking Union Medical College, Chinese Academy of Medical Science, Beijing, China; 4 Department of Laboratory Medicine, Peking University Third Hospital, Peking University, Beijing, China; 5 Department of Dermatology and Venerology, Peking University First Hospital, Peking University, Beijing, China; 6 Department of Laboratory Medicine, The First Affiliated Hospital of Sun Yat-sen University, Guangzhou, China; 7 Union Hospital Tongji Medical College of Huazhong University of Science and Technology, Wuhan, China; 8 The First Hospital of Jilin University, Jilin, China; 9 Department of Laboratory Medicine, The First Affiliated Hospital of Xinjiang Medical University, Wulumuqi, China; 10 Department of Microbiology, The First Affiliated Hospital of Zhejiang University, Hangzhou, China; 11 The First Affiliated Hospital of Chongqing Medical University, Chongqing, China; 12 Department of Laboratory Medicine, Lishui Municipal Central Hospital, Lishui, China; 13 Biomedical Engineering Facility of National Infrastructures for Translational Medicine, Peking Union Medical College Hospital, Beijing, 100730 China

**Keywords:** *Candida Glabrata*, Echinocandin resistance, Whole genome sequence

## Abstract

**Background:**

*Candida glabrata* is an important cause of invasive candidiasis. Echinocandins are the first-line treatment of invasive candidiasis caused by *C. glabrata*. The epidemiological echinocandin sensitivity requires long-term surveillance and the understanding about whole genome characteristics of echinocandin non-susceptible isolates was limited.

**Results:**

The present study investigated the echinocandin susceptibility of 1650 *C. glabrata* clinical isolates in China from August 2014 to July 2019. The in vitro activity of micafungin was significantly better than those of caspofungin and anidulafungin (*P* < 0.001), assessed by MIC_50/90_ values. Whole genome sequencing was conducted on non-susceptible isolates and geography-matched susceptible isolates. Thirteen isolates (0.79%) were resistant to at least one echinocandin. Six isolates (0.36%) were solely intermediate to caspofungin. Common evolutionary analysis of echinocandin-resistant and echinocandin-intermediate isolates revealed genes related with reduced caspofungin sensitivity, including previously identified sphinganine hydroxylase encoding gene *SUR2*. Genome-wide association study identified SNPs at subtelometric regions that were associated with echinocandin non-susceptibility. In-host evolution of echinocandin resistance of serial isolates revealed an enrichment for non-synonymous mutations in adhesins genes and loss of subtelometric regions containing adhesin genes.

**Conclusions:**

The echinocandins are highly active against *C. glabrata* in China with a resistant rate of 0.79%. Echinocandin non-susceptible isolates carried common evolved genes which are related with reduced caspofungin sensitivity. In-host evolution of *C. glabrata* accompanied intensive changing of adhesins profile.

**Supplementary Information:**

The online version contains supplementary material available at 10.1186/s12866-023-03105-3.

## Background

*Candida glabrata* is a component of the human microbiome and is a prevalent opportunistic fungal pathogen causing bloodstream infection that have high mortality rates [1]. In countries like USA and Australia, *C. glabrata* is the second most common cause of candidemia after *Candida albicans* [2, 3]. The number of candidemia cases caused by *C. glabrata* exhibits a temporal increasing trend [4]. The increasing burden of fungal infections has led to rising usage of antifungal agents for their treatment and prevention. *C. glabrata* is known to exhibit reduced susceptibility or resistance to fluconazole and the other azoles [5, 6]. Global azole resistance among *C. glabrata* isolates is around 8% [7], while some centers report rates exceeding 20% [8]. The innately low susceptibility to azole drugs of *C. glabrata* has led to the widespread use of echinocandin antifungal drugs.

The echinocandins (anidulafungin, micafungin, and caspofungin), which target and inhibit the membrane-associated β-1-3-D-glucan synthase, are considered fungicidal drugs against *Candida* species. According to large-scale surveillance studies, the overall prevalence of *C. albicans* resistance is less than 1% and resistance among most susceptible *Candida* species is at or below this value [9]. While for *C. glabrata*, most epidemiological prevalence studies report echinocandin resistance of 2–4% [10]. Echinocandin use has expanded in the past decade, which has increased the potential for the emergence of antimicrobial resistance [11]. In a population-based candidemia surveillance study, the proportion of non-susceptible isolates increased from 4.2% to 2008 to 7.8% in 2014 [12]. According to the China Hospital Invasive Mycosis Surveillance Network (CHIF-NET), the proportion of echinocandin-resistant *C. glabrata* isolates during 2009–2014 was 0.5% [13]. The mechanism of echinocandin resistance in *Candida* species involves genetic acquisition of mutations in *FKS* genes, which encode the catalytic subunits of glucan synthase [14]. In *C. glabrata*, amino acid substitutions in both Fks1 and Fks2 occur, but are more common in Fks2 [15]. Besides the well-known echinocandin-resistant mechanism of Fks mutation, some genes had been reported to be related with reduced sensitivity to caspofungin, like *SUR2*, a sphinganine hydroxylase with role in sphingolipid biosynthesis [16]. Some zinc cluster proteins which involved in regulation of many cellular processes also participated in drug resistance of fungi [17]. Given the importance of this drug class as a first-line agent, there is an urgent need to better monitor the epidemiological antifungal sensitivity data and understand the factors that contribute to the emergence of echinocandin resistance.

The present study reports the echinocandin sensitivity of *C. glabrata* isolates collected by CHIF-NET program from 82 hospitals, 26 provinces in China, from August 2014 to July 2019. Comparative genomic analysis was performed on echinocandin non-susceptible isolates and geography-matched echinocandin-susceptible isolates. Besides that, in-host evolutionary changes between serial isolates which acquired echinocandin resistant during treatment were analyzed.

## Results

### Clinical information and echinocandin susceptibility of clinical isolates

**Fig. 1:**
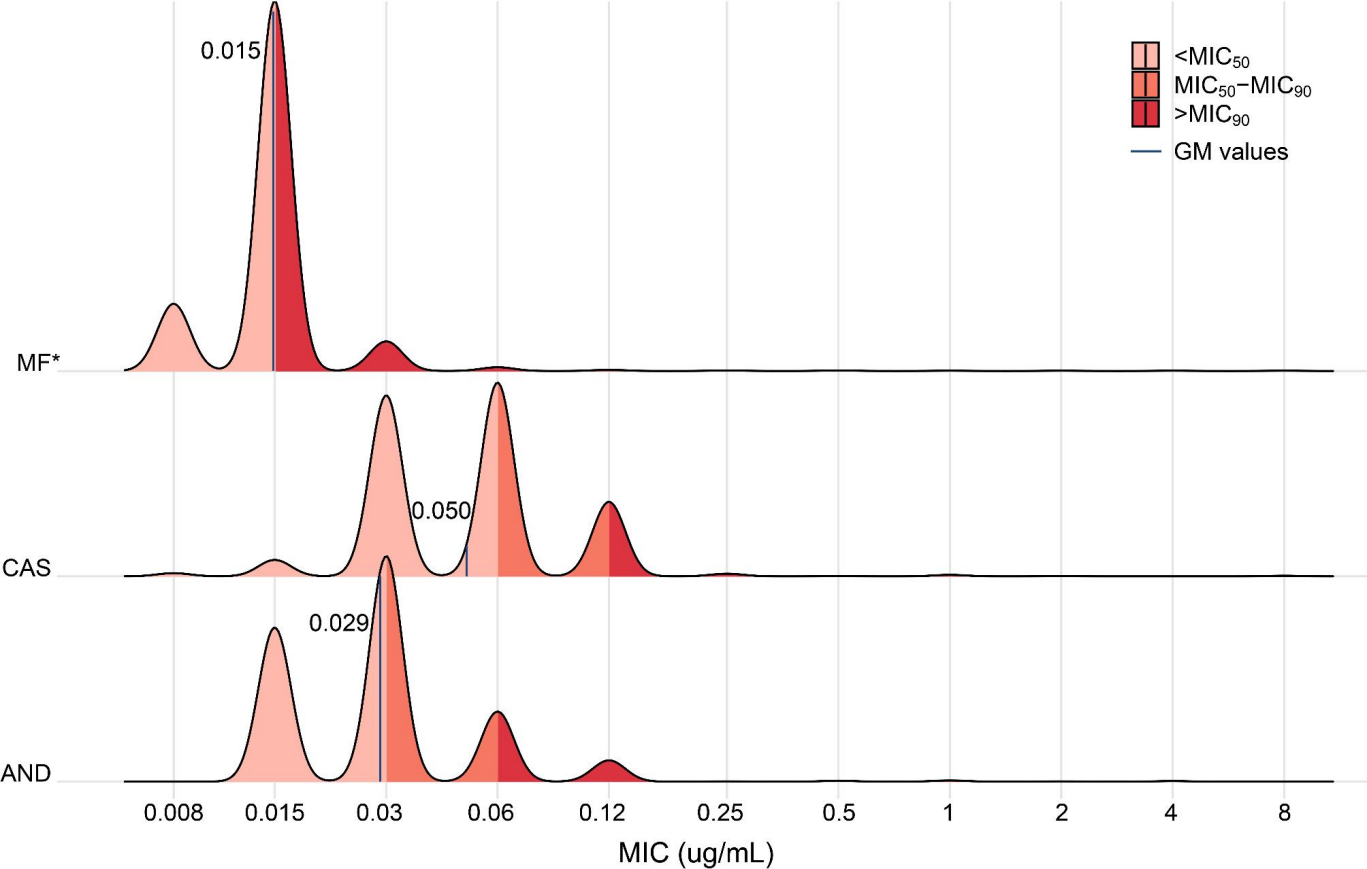
Echinocandin susceptibility of *Candida glabrata* clinical isolates. *, *P* < 0.001

Totally 1650 *C. glabrata* clinical isolates were collected from 82 hospitals, 26 provinces in China from August 2014 to July 2019. Most of these strains were isolated from ICU (33.52%, 553/1650), surgical ward (32.42%, 535/1650), and internal medicine ward (19.94%, 329/1650). Of various specimen types, these strains were mainly recovered from blood (46.91%, 774/1650) and ascites (14.18%, 234/1650). Among three echinocandins tested, caspofungin exhibited the highest MIC levels (geometric mean MIC (GM): 0.05 µg/mL; MIC_50/90_: 0.06/0.12 µg/mL) (Fig. 1). The in vitro activity of micafungin was significantly better than those of the other two echinocandins (*P* < 0.001), with MIC_50/90_ values of 0.015/0.015 µg/mL and a GM of 0.015 µg/mL (Fig. 1).

Among the 1650 isolates, 99.33%, 99.27%, and 98.91% were susceptible to anidulafungin, micafungin, and caspofungin, respectively. Thirteen isolates (0.79%, 13/1650) were resistant to at least one echinocandin but all of them were sensitive to fluconazole. No multidrug resistance was noticed. Six isolates (0.36%, 6/1650) were susceptible to anidulafungin and micafungin, but intermediate to caspofungin (MIC: 0.25 µg/mL) with one of them was resistant to fluconazole (MIC: 256 µg/mL). No differences were found between echinocandin non-susceptible and susceptible isolates in patients’ age, sex, and specimen types (Table S1). The echinocandin non-susceptible rates of isolates from ICU (1.81%, 10/553) and surgical ward (1.31%, 7/535) were significantly higher than that of isolates from internal medicine ward (0%, 0/329; *P* = 0.034 when compared with ICU; *P* = 0.048 when compared with surgical ward).

### Whole genome sequencing and distribution of sequence types

To investigate the genetic characteristics of echinocandin non-susceptible isolates, the genomes of 19 echinocandin non-susceptible isolates and 35 geography-matched echinocandin susceptible isolates were sequenced and analyzed. Clinical information of these isolates was provided in Table S2.

By WGS, there were 11 distinct sequence types (STs) defined based on the alleles from six genetic loci (*FKS, LEU2, NMT1, TRP1, UGP1*, and *URA3*), including 1 new ST (herein assigned as N1) not recognized by the *C. glabrata* MLST database (https://pubmlst.org/cglabrata/). The commonest ST in these isolates was ST7 (53.73%, 29/54) followed by ST10 (11.11%, 6/54), ST3 (9.26%, 5/54), and ST19 (9.26%, 5/54). The difference of the echinocandin non-susceptible rates between ST7 isolates and non ST7 isolates was not significant (41.38% vs. 28%, *P =* 0.304).

### Phylogenetic analysis and genes related with echinocandin non-susceptibility

**Fig. 2:**
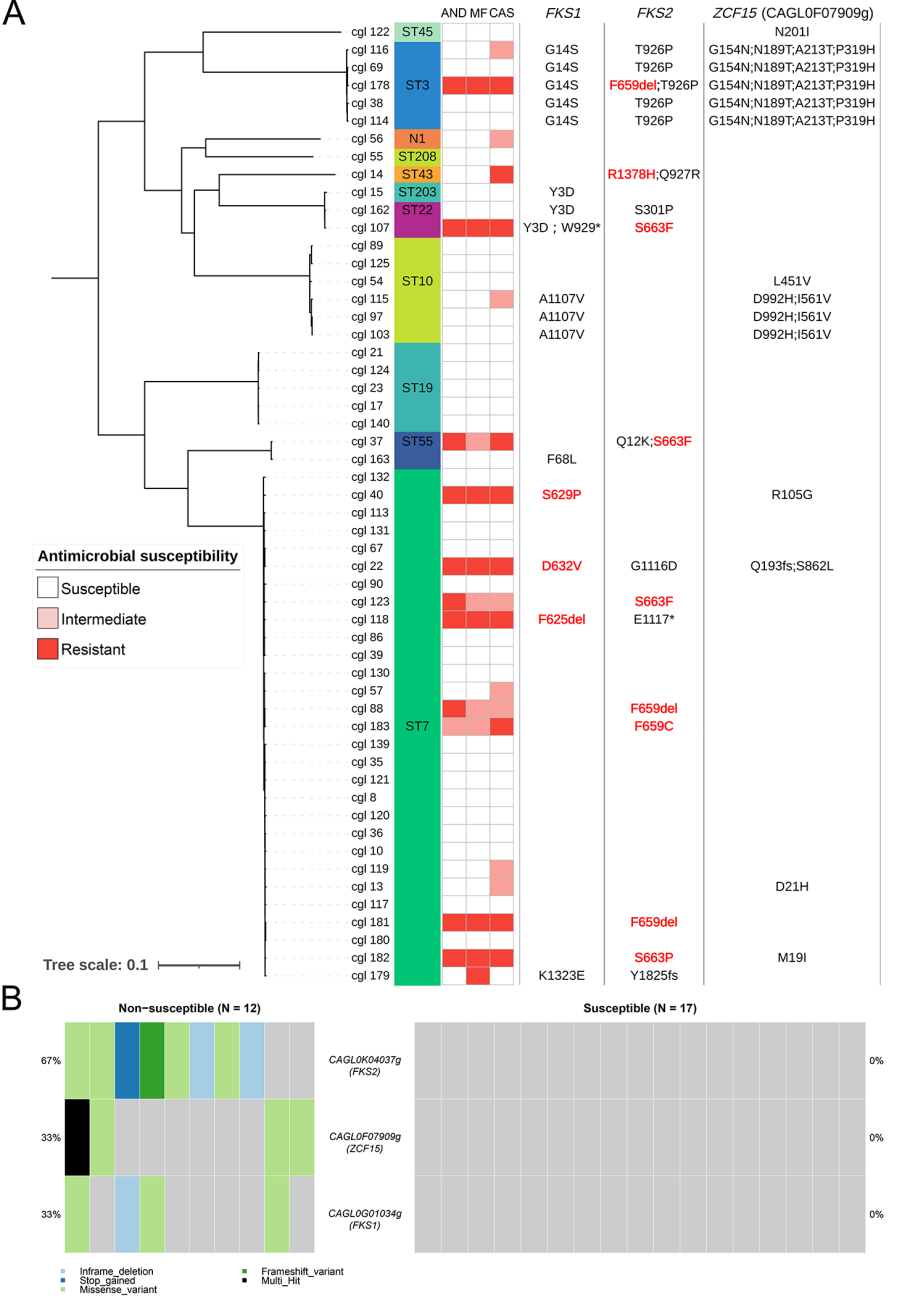
Maximum-likelihood tree of 54 isolates based on whole genome sequences (**A**) and genes (*FKS2*, *ZCF15*, and *FKS1*) that had significantly higher mutation rates in echinocandin non-susceptible isolates of ST7 (**B**). Echinocandin susceptibility and amino acid substitution/deletion of each isolate were annotated. Well-known *FKS* hotspot mutations were colored in red

In general, the whole genome SNP phylogenetic tree clustered broadly within determined STs (Fig. 2A). Totally 568,272 (10,523.56 per isolates) nonsynonymous mutations and indels were identified in 4,711 genes. Then, the mutation rates of each gene were compared between echinocandin-susceptible and non-susceptible isolates. To eliminate the influence of the genetic differences among different STs, only 29 isolates of ST7 (12 non-susceptible and 17 susceptible isolates) were analyzed further in detail. The result showed that 3 genes (*FKS2*, *ZCF15*, and *FKS1*) had significantly higher mutation rates in echinocandin non-susceptible isolates than in susceptible isolates (Fig. 2B). *ZCF15* (*CAGL0F07909g*), which encodes a zinc ion binding protein which is involved in regulation of transcription, is a newly identified gene which is related with echinocandin non-susceptibility.

The amino acid changes in these three genes of all isolates were shown in Fig. 2A (right panel). Mutations *FKS1*^G14S^ and *FKS2*^T926P^ seem specific to ST3. *FKS2*^F659C/del^ (30.77%, 4/13) and *FKS2*^S663P/F^ (30.77%, 4/13) are most common mutations detected in echinocandin-resistant isolates. All but one echinocandin-resistant isolates carried typical mutations in hotspot regions of *FKS1* or *FKS2*. Strain cgl_179, which is susceptible to anidulafungin and caspofungin but resistant to micafungin (MIC 0.5 µg/mL), carries *FKS1*^K1323E^ and frame shift variation *FKS2*^Y1825fs^. In addition, all six isolates which are only intermediate to caspofungin do not carry mutations in hotspot regions of *FKS* genes. Among isolates of ST7, nonsynonymous mutations in *ZCF15* were detected in three echinocandin-resistant isolates and one echinocandin-intermediate isolate.

### Common evolved genes in echinocandin-intermediate and echinocandin-resistant isolates

**Fig. 3:**
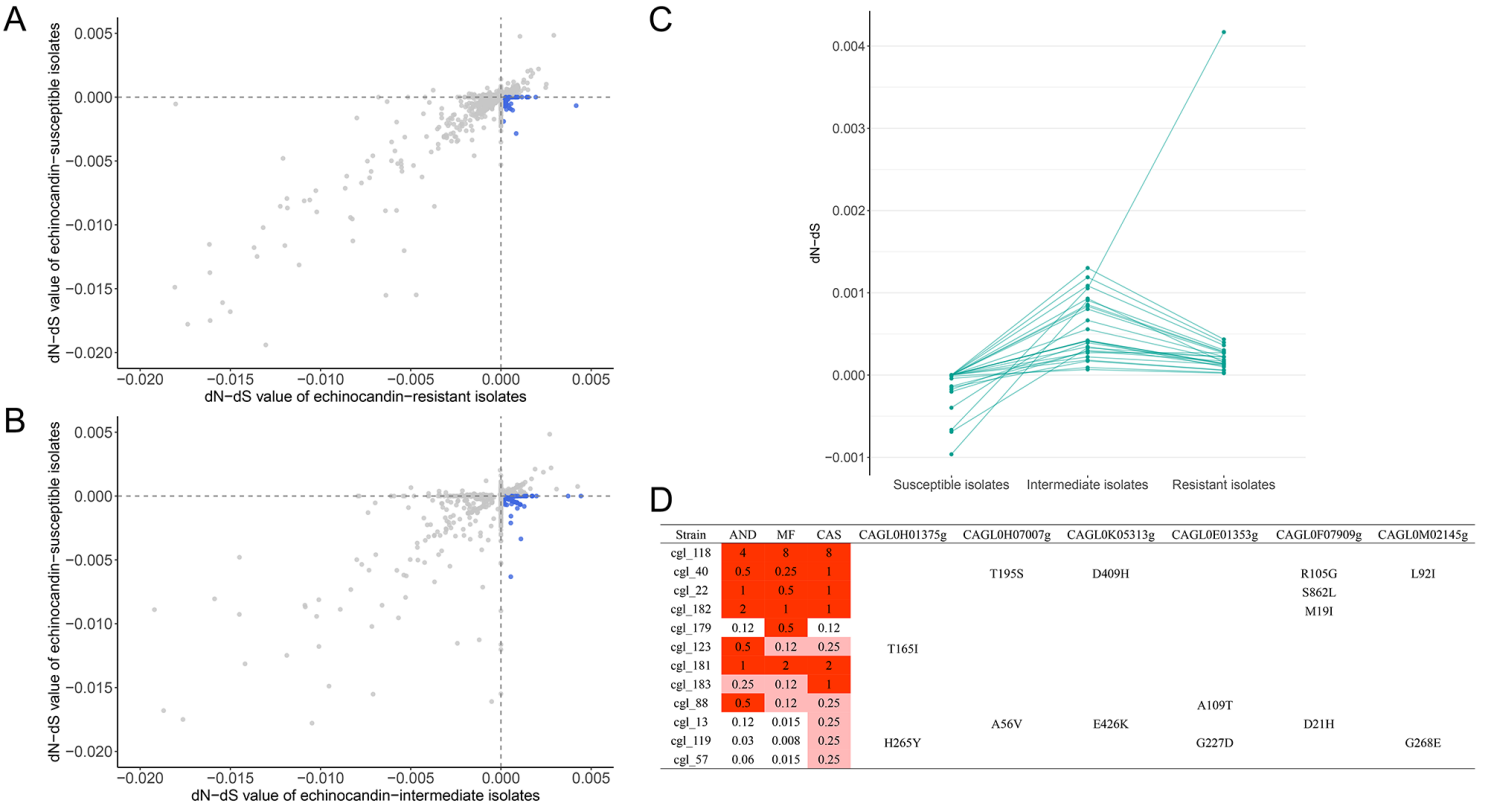
The dN-dS values of all annotated genes of *Candida glabrata* between echinocandin susceptible and resistant isolates (**A**), and that between echinocandin susceptible and intermediate isolates (**B**). The dN-dS values of common evolved genes in echinocandin intermediate and resistant isolates (**C**). Amino acid substitutions of genes with nonsynonymous mutations only detected in echinocandin non-susceptible isolates (**D**)

To figure out genes related with reduced echinocandin sensitivity in echinocandin-intermediate isolates, the dN-dS values for all annotated genes of *C. glabrata* were calculated to investigate the common evolved genes in echinocandin-intermediate and resistant isolates. In order to eliminate the influence of the genetic differences among different STs, only 29 isolates of ST7 were analyzed. We selected genes which are under positive selection (dN-dS > 0) in echinocandin-resistant isolates but not in echinocandin-susceptible isolates (Fig. 3A). Similarly, genes which are under positive selection in echinocandin-intermediate isolates but not in echinocandin-susceptible isolates were also selected (Fig. 3B). The intersection of these two gene sets contains 26 common evolved genes in echinocandin-intermediate and echinocandin-resistant isolates (Fig. 3C). The descriptions of these genes were searched in the Candida Genome Database [18] and provided in Table S3. Functions of these gene mainly includes sphingolipid biosynthetic process, protein retention in Golgi apparatus and regulation of transcription. Nonsynonymous mutations of these genes were manually scanned and analyzed. There were six genes with nonsynonymous mutations only detected in echinocandin non-susceptible isolates (Fig. 3D). *SUR2* (*CAGL0H01375g*) is a sphinganine hydroxylase with role in sphingolipid biosynthesis. Mutants of this gene show reduced sensitivity to caspofungin [16]. Mutations *SUR2*^T165I^ and *SUR2*^H265Y^ found in non-susceptible isolates were in the fatty acid hydroxylase domain (162–297) which could impact the function of this protein. As mentioned before, *ZCF15* (*CAGL0F07909g*) encodes a zinc ion binding protein which is involved in regulation of transcription. Mutations *ZCF15*^M19I^ and *ZCF15*^D21H^ were in the DNA-binding domain (14–43) which could impact the function of this protein. Other four genes were not characterized in *C. glabrata*. The *S. cerevisiae* ortholog of these genes encodes protease (*CAGL0H07007g*), histidyl-tRNA synthetase (*CAGL0K05313g*), zinc transporter of the plasma membrane (*CAGL0E01353g*), and nucleolar RNA methyltransferase (*CAGL0M02145g*).

### Genome wide association study

**Fig. 4:**
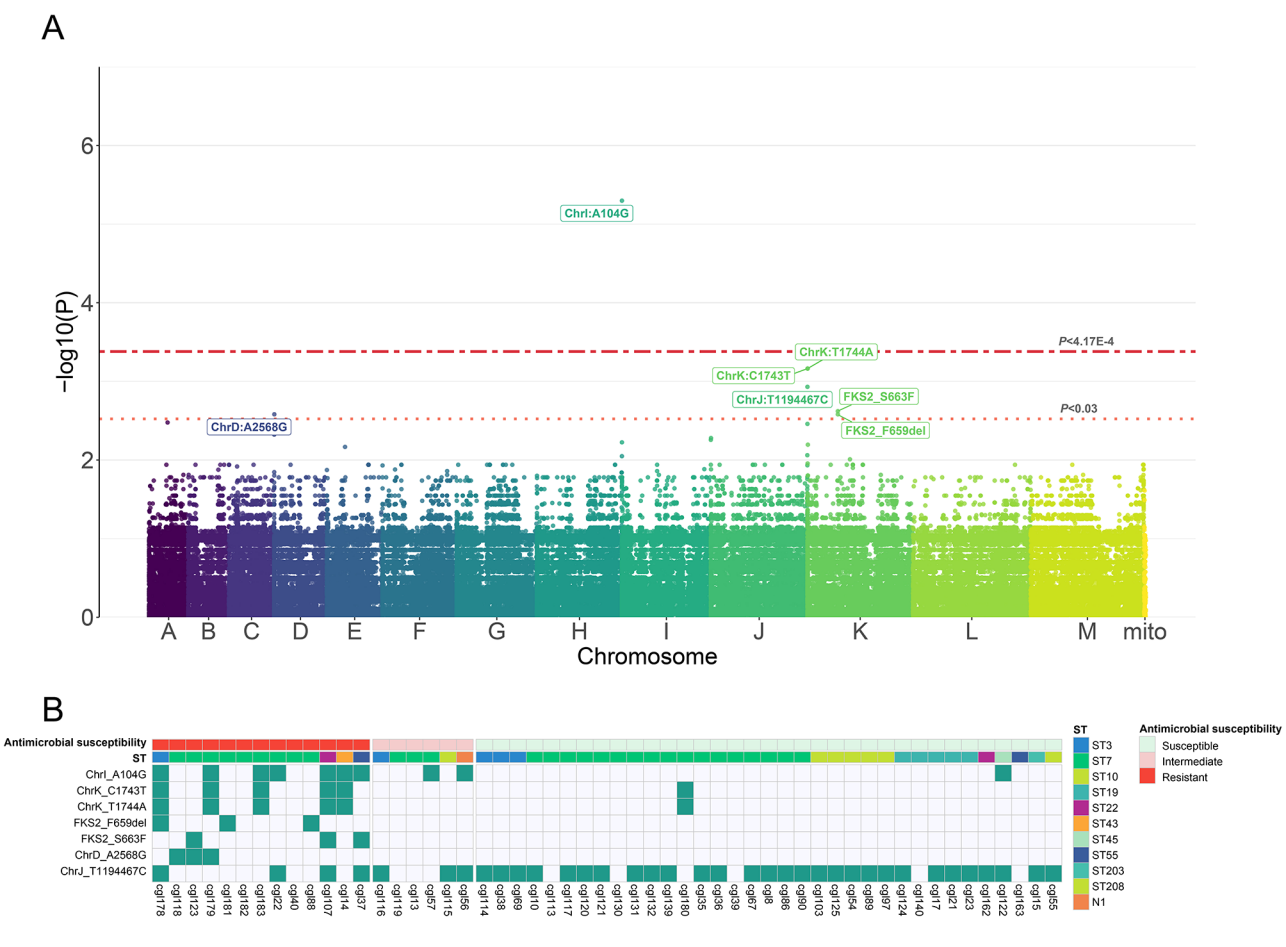
Manhattan plot of the GWAS analysis for association between genotypes and echinocandin susceptibility

Given that mutations in intergenic regions could also have influence on phenotype changes. Genome wide association study (GWAS) was conducted to identify genetic variants that contribute to echinocandin non-susceptibility (Fig. 4A). All 54 genomes were included in the GWAS using a univariate linear mixed model (LMM) approach which can correct sample relatedness and population stratification [19]. After removing low frequency sites, we tested 246,053 SNPs and identified one SNP having significant association signals after multiple test correction (*P* < 4.17E-4). This mutation lies in the subtelometric region at the start of chromosome I (A104G). It was detected in 53.85% (7/13) of echinocandin-resistant isolates, 33.33% (2/6) of echinocandin-intermediate isolates, and 2.86% (1/35) of echinocandin-susceptible isolates (Fig. 4B). The cutoff *P* by Bonferroni correction (4.17E-4) [20] could be strict because known echinocandin-resistant mutations (*FKS2*^S663F^ and *FKS2*^F659del^) were also identified with *P* < 0.03 (Fig. 4A). Besides these two classic mutations, all the other 6 mutations with *P* < 0.03 were located at subtelometric regions. The co-existence of A104G of chromosome I, and C1743T and T1744A of chromosome K was found in 38.46% (5/13) of echinocandin-resistant isolates.

### Genetic changes of ***Candida glabrata*** in-host evolution from echinocandin-susceptible to echinocandin-resistant

**Fig. 5:**
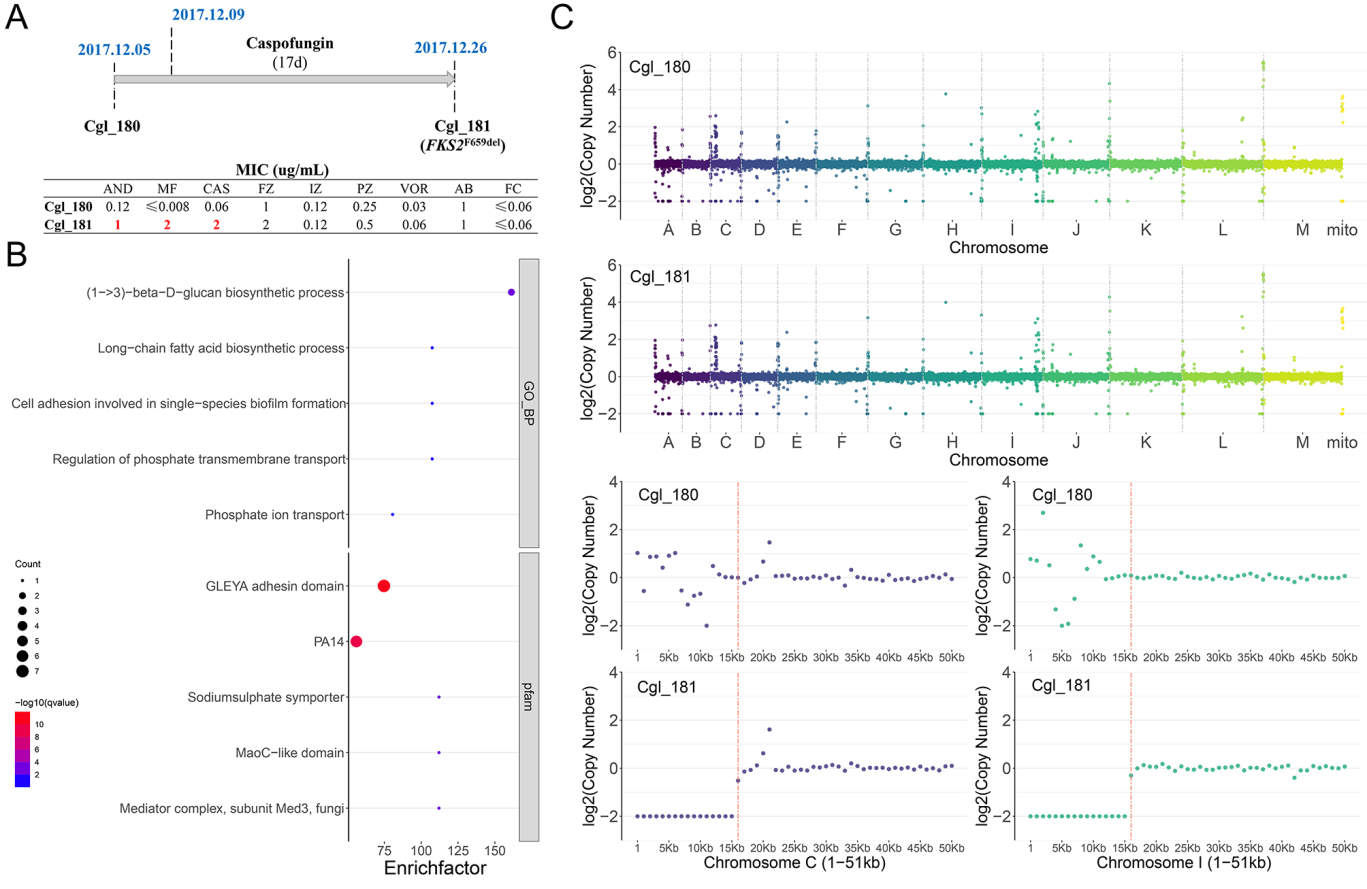
Clinical information of serial isolates (**A**). GO and Pfam annotation of genes with non-synonymous mutation between serial isolates (**B**). Copy number variations of serial isolates (**C**)

Strains cgl_180 and cgl_181 are serial isolates from the bloodstream of an 80 years old female obtained at a 21-day interval (Fig. 5A). The patient was treated with caspofungin for 17 days before pan-echinocandin resistant strain cgl_181 was isolated from her blood. SNP and CNV of these two isolates were compared. Besides *FKS2*^F659del^, nonsynonymous mutations were also detected in other 42 genes in cgl_181 compared to cgl_180. GO annotations of these genes were weakly enriched in β-1-3-D-glucan and fatty acid biosynthetic process (Fig. 5B). Pfam domains of these genes were enriched in GLEYA adhesin and PA14 domain, which can also be found in yeast adhesins (Fig. 5B). To be specific, non-synonymous mutations were detected in epithelial adhesins (*EPA*) 2, *EPA3*, *EPA8*, *EPA9*, *EPA11*, *EPA12*, and *EPA15*. CNV of whole genomes between these two isolates shows similar pattern with little difference found at the start of Chromosome C and I (Fig. 5C). Compared with cgl_180, 15 kb deletions were found in Chromosome C and I of strain cgl_181 (Fig. 5C). These regions contain adhesins *EPA6* (*CAGL0C00110g*), *AWP7* (*CAGL0C00209g*) and putative adhesin *CAGL0I00209g*. These results indicate the fast evolution of adhesins of *C. glabrata* during blood stream infection.

## Discussion

Compared to our previous study (the CHIF-NET 2009–2014 program) [13], which collected 411 *C. glabrata* isolates from 11 hospitals in 8 provinces, the present study (the CHIF-NET 2015–2019 program) collected 1650 isolates from 82 hospitals in 26 provinces in China. With more participants from more geographic regions, the data of the present study would be more convincing.

According to the global SENTRY study, the incidence of echinocandin-resistant *C. glabrata* isolates during 2006–2016 was 1.7–3.5% [21]. China had relatively lower resistance rate. We previously reported the proportion of echinocandin-resistant *C. glabrata* isolates from August 2009 to July 2014 was 0.5% [13], and the proportion increased to 0.79% from August 2014 to July 2019. This is consistent with the result of a systematic review in China, which reports the echinocandin resistance of 0.8–2.5% during 2011–2021 [22]. Among the tested echinocandins, micafungin showed significantly better in vitro activity against the *C. glabrata* isolates than both anidulafungin and caspofungin, which is consistent with the results of the SENTRY Antifungal Surveillance Program [21]. The prevalence of different STs varies by geographic regions. ST5 isolates were reportedly common in Europe [23]. Strains of ST8, ST18, and ST19 were the commonest types in the United States [23], while ST7 are more prevalent in Korea [24] and Japan [23]. Consistent with our previous study [13], ST7 is still the most prevalent ST in China. The echinocandin sensitivity among different STs were similar. Although 12 out of 19 non-susceptible isolates were belong to ST7, the difference of the echinocandin non-susceptible rates between ST7 isolates and non-ST7 isolates was not significant. Due to the limitation of sample size in this study, further studies are needed to investigate the antimicrobial profiles of different STs.

The mechanism of echinocandin resistance in *Candida* species involves genetic acquisition of mutations in *FKS* genes [14]. S629P in Fks1, and S663P and F659deletion in Fks2 are the most prominent substitutions involved in both in vitro and in vivo resistance [4]. Besides the known hotspot mutations in *FKS1* and *FKS2*, mutations occurring outside of these hotspot regions can also lead to echinocandin resistance. We previously reported a pan-echinocandin resistant isolate carries an E655K mutation just upstream of the hotspot region of *FKS2* and a premature stop codon in *FKS1* [25]. Similarly, the present study identified a new variant harboring *FKS1*^K1323E^ and *FKS2*^Y1825fs^, which is solely resistant to micafungin. Both mutations located outside the hotspot regions and their role in micafungin resistance need further in vitro and in vivo investigations.

Whole genome sequencing on *C. glabrata* clinical isolates was performed by some studies to investigate the genomic diversity on this species [26–29]. However, no comparative genomic study was performed on echinocandin-resistant isolates. The present study identified some genomic characteristics in echinocandin non-susceptible isolates. There were six genes might have relationship with reduced sensitivity to caspofungin. Previous study found mutations of *SUR2* (*CAGL0H01375g*), a sphinganine hydroxylase with role in sphingolipid biosynthesis, could lead to reduced sensitivity to caspofungin [16]. The author speculated the disruption of sphingolipid biosynthesis pathway led to the accumulation of long-chain bases dihydrosphingosine or phytosphingosine, which could further weaken the interaction between the membrane-spanning hotspot regions of Fks and the lipid tail of caspofungin. *ZCF15* (*CAGL0F07909g*) encodes a zinc ion binding protein. Zinc cluster proteins of fungi involved in regulation of many cellular processes such as the metabolism of amino acids, carbon (sugars and nonfermentable carbon sources), pyrimidine, fatty acid, as well as drug resistance [17, 30]. The role of *ZCF15* and other uncharacterized genes in reduced caspofungin sensitivity need further investigation.

*C. glabrata* has been reported to have a highly dynamic genome under clinical conditions, especially in subtelometric regions [28, 31]. This may be due to its asexual nature and haploid chromosomes. Except well-known SNPs in *FKS2*, we identified five SNPs that are significantly associated with echinocandin susceptibility and all of them were in subtelometric regions, which contain numerous epithelial adhesin (EPA) genes [32, 33]. The potential role of these mutations in echinocandin resistance needs further investigation. In-host evolutionary changes between serial isolates were enriched in adhesin-like proteins which is consistent with previous study [27], suggesting that EPA genes are undergoing variation during in-host evolution. As EPA also involved in host cell recognition of *C. glabrata*, the variation of EPA might be immune evasion mechanism during in-host evolution [34]. In addition, the CNVs observed between serial isolates reflect the shortening of telomere length during the acquirement of echinocandin resistance. It has been reported that the telomere length of drug-resistant cancer cells shortened under the chemotherapeutic stress [35]. The relationship between shortening of telomere and echinocandin resistance needs further investigation.

## Conclusion

The present study reports the echinocandin susceptibility of *C. glabrata* in China from August 2014 to July 2019 and explored genomic variations related with echinocandin non-susceptibility, as well as changing genetic characteristics during in-host evolution. Future studies are warranted to investigate the relationship between reduced echinocandin sensitivity and genetic variants identified in the present study.

## Methods

### Isolates and identification

*Candida glabrata* isolates were collected prospectively over the 5-year study period from patients enrolled in the CHIF-NET study, a laboratory-based, national multicenter surveillance program conducted during August 2014 to July 2019. Isolates were identified as *C. glabrata* by a previously-established algorithm incorporating matrix-assisted laser desorption ionization-time of flight mass spectrometry (MALDI-TOF MS) (Vitek MS, bioMérieux, Marcy l’Etoile, France) supplemented with rDNA internal transcribed spacer (ITS) sequencing [36]. In comparative genomic analysis, in order to investigate the genetic characteristics of echinocandin non-susceptible isolates, the genomes of 19 echinocandin non-susceptible isolates and 35 geography-matched echinocandin-susceptible isolates, including a serial isolate of an echinocandin-resistant isolate, were enrolled. Clinical information of these isolates was provided in Table S2.

### Susceptibility testing

Susceptibility to antifungal agents were determined using the Sensititre YeastOne™ YO10 methodology (Thermo Scientific, Cleveland, OH, USA) according to Clinical and Laboratory Standards Institute (CLSI) methodology. *Candida parapsilosis* ATCC 22019 and *Candida krusei* ATCC 6258 were used as quality control. MIC values were interpreted according to CLSI M60 guidelines for fluconazole and the echinocandins [37].

### Library preparation for whole genome sequencing

The sequencing was conducted by OE Biotech Co., Ltd. (Shanghai, China). The libraries were constructed with TruSeq Nano DNA LT Sample Prepararion Kit (Illumina, San Diego, CA, USA). Briefly, the genomic DNA was sheared into fragments with length ~ 350 bp using S220 Focused-ultrasonicators (Covaris, USA). Adapters were ligated onto the 3’ end of the sheared fragments. After PCR amplification and purification, the final libraries were sequenced on the Illumina sequencing platform HiSeq X Ten platform (Illumina Inc., San Diego, CA, USA) and 150 bp paired-end reads were generated.

### Whole genome sequencing data analysis

The raw reads were subjected to a quality check and then filtered by fastp (Version 0.19.5) [38]. Clean reads were aligned to the reference genome of *C. glabrata* CBS138 (http://www.candidagenome.org/download/sequence/C_glabrata_CBS138/archive/C_glabrata_CBS138_version_s03-m01-r26_chromosomes.fasta.gz) using Burrows-Wheeler Aligner (BWA, Version 0.7.12) [39]. After alignment, Picard (http://broadinstitute.github.io/picard/, Version 4.1.0.0) was employed to mark duplicate reads. The Genome Analysis Toolkit (GATK) v.4.1.2.0 [40] was used to call variants. Then SnpEff [41] was applied to annotate all the variants. The copy number variations (CNVs) were identified using HMMcopy based on the ReadDepth method [42]. A sliding window (1 kb) approach was used to determine Reads Depth. The direction and magnitude of natural selection for each gene were assessed by measuring the value of the nonsynonymous substitution (dN) minus the synonymous substitution (dS) using MEGAX [43].

### Multi locus sequence types and phylogenetic analysis

In silico MLST sequence types (STs), inferred from whole genome sequence data (genome types) were obtained from assembled contigs using SPAdes [44] and MLST software (https://github.com/tseemann/mlst). To infer the phylogenetic relationship of the isolates, the best-fitting substitution model (TVM + F) was selected with the Bayesian Information Criterion using Model Finder implemented in IQ-Tree v.1.6.2 [45]. Then, a maximum likelihood tree was reconstructed using IQ-Tree using 1000 ultrafast bootstrap replicates. The phylogenetic tree was visualized using iTOL tree [46].

### Genome-wide association study

Genome-wide association study (GWAS) was performed with R package GEMMA (version 0.94beta) using a univariate linear mixed model (LMM) approach which can correct sample relatedness and population stratification [19]. The p-value cutoff by Bonferroni correction for family-wise error rate 0.05 was generated using GEC software [20].

### Statistical analyses

Categorical variables were expressed as % (m/n) and examined using χ^2^/Fisher’s exact test. Non-normally distributed data were expressed as median and interquartile range and compared using Mann-Whitney U-test. All *P* values were two-tailed and a *P* value < 0.05 was considered statistically significant. Statistical analyses were performed and graphs were plotted using R (4.2.1) (https://cran.r-project.org).

### Electronic supplementary material

Below is the link to the electronic supplementary material.

Supplementary Material 1

## Data Availability

All short-read data were uploaded to the NCBI under the project number PRJNA1010673 (https://www.ncbi.nlm.nih.gov/bioproject/PRJNA1010673).

## Abbreviations

STs: Sequence types
SNP: Single nucleotide polymorphism
CNV: Copy number variation
