# Correction: Whole genome analysis of echinocandin non-susceptible *Candida Glabrata* clinical isolates: a multi-center study in China

**DOI:** 10.1186/s12866-023-03130-2

**Published:** 2023-11-30

**Authors:** Yi Li, Xin Hou, Ruoyu Li, Kang Liao, Ling Ma, Xiaoming Wang, Ping Ji, Haishen Kong, Yun Xia, Hui Ding, Wei Kang, Ge Zhang, Jin Li, Meng Xiao, Yingxing Li, Yingchun Xu

**Affiliations:** 1 Department of Laboratory Medicine, State Key Laboratory of Complex Severe and Rare Diseases, Peking Union Medical College Hospital, Chinese Academy of Medical Sciences and Peking Union Medical College, Beijing, China; 2 Beijing Key Laboratory for Mechanisms Research and Precision Diagnosis of Invasive Fungal Diseases, Beijing, China; 3 Graduate School, Peking Union Medical College, Chinese Academy of Medical Science, Beijing, China; 4 Department of Laboratory Medicine, Peking University Third Hospital, Peking University, Beijing, China; 5 Department of Dermatology and Venerology, Peking University First Hospital, Peking University, Beijing, China; 6 Department of Laboratory Medicine, The First Affiliated Hospital of Sun Yat-sen University, Guangzhou, China; 7 Union Hospital Tongji Medical College of Huazhong University of Science and Technology, Wuhan, China; 8 The First Hospital of Jilin University, Jilin, China; 9 Department of Laboratory Medicine, The First Affiliated Hospital of Xinjiang Medical University, Wulumuqi, China; 10 Department of Microbiology, The First Affiliated Hospital of Zhejiang University, Hangzhou, China; 11 The First Affiliated Hospital of Chongqing Medical University, Chongqing, China; 12 Department of Laboratory Medicine, Lishui Municipal Central Hospital, Lishui, China; 13 Biomedical Engineering Facility of National Infrastructures for Translational Medicine, Peking Union Medical College Hospital, Beijing, 100730 China

**Correction: BMC Microbiol 23, 341 (2023)**

10.1186/s12866-023-03105-3

Following publication of the original article [1], the author reported that this article should have two corresponding authors: Yingxing Li and Yingchun Xu, as they mentioned in the original and revised manuscript. However, it is only Yingxing Li who is captured as corresponding author in the published version. Xu Yingchun’s email address is xycpumch@139.com.

The original article [1] has been corrected.
